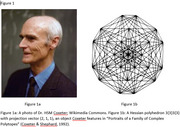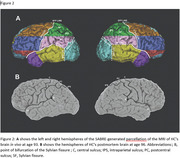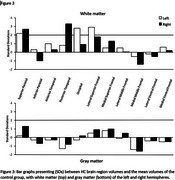# Brain Morphology in Extraordinary Geometrician Harold Coxeter: implications for connectivity

**DOI:** 10.1002/alz70857_107442

**Published:** 2025-12-26

**Authors:** Christopher JM Scott, Debra Kigar, Pei Harry Li, Aaron Wen, Devon Malhotra, Zain Daudi, Sandra E. Black, Sandra F Witelson

**Affiliations:** ^1^ Dr. Sandra Black Centre for Brain Resilience & Recovery, Sunnybrook Research Institute, Toronto, ON, Canada; ^2^ Department of Psychiatry and Behavioural Neurosciences, McMaster University, Hamilton, ON, Canada; ^3^ Department of Psychiatry and Behavioural Neurosciences, McMaster University, Hamilton, ON, Canada; ^4^ Dr. Sandra Black Centre for Brain Resilience & Recovery, Toronto, ON, Canada

## Abstract

**Background:**

While extensive research has examined brain‐behavior relationships in cognitive decline, far less study of the other extreme has been done with super‐agers or those with extraordinary abilities. Harold Coxeter (HC), an extraordinary geometrician (Figure 1), considered one of the foremost mathematical minds of the 20th century, volunteered to have his brain studied after learning about the neuroanatomical analysis of Albert Einstein's brain (Witelson et al., 1999). We aimed to explore whether HC's exceptional geometrical prowess was related to variations in brain anatomy, particularly the parietal lobes.

**Method:**

At the age of 93, HC underwent structural MRI, which was analyzed using Semi‐Automated Brain Region Extraction (SABRE) and FreeSurfer (Figure 2). Brain images were compared to 24 neurotypical men of senior age. Grey matter (GM) and white matter (WM) volumes were assessed in HC and controls, focusing on key regions associated with mathematical cognition and spatial abilities.

**Result:**

No significant differences in GM volumes were observed between HC and controls in any region, based on both SABRE and FreeSurfer analyses. However, HC exhibited larger WM volumes in several brain regions, notably in the right and left superior parietal regions, as well as the left superior frontal, left occipital, and right posterior temporal regions, where HC's white matter volume exceeded the control group mean by at least two standard deviations (Figure 3). Moreover, his WM volumes were greater than that of any individual control.

**Conclusion:**

The findings suggest that HC's exceptional abilities may be associated with increased connectivity mediated by WM tracts in specific brain regions, particularly those related to visuospatial processing and mathematical cognition. These results support the general hypothesis that WM connectivity may play a key role in intelligence variability, especially in domains involving spatial and mathematical processing. We found no evidence for GM differences. Some other studies of cognitive expertise showed increased GM density, but used transformed measures of relative GM density, whereas our method allows direct quantification of tissue volumes. This study highlights the potential link between structural brain differences and variations in cognitive ability, offering insight into the neurobiological basis of some aspects of human intelligence.